# Design and Experiment Investigation on Soft Grippers with Modular Variable Stiffness Structure

**DOI:** 10.3390/mi15010088

**Published:** 2023-12-30

**Authors:** Pengbing Zhao, Chuan Xiong, Zheng Gao, Xiang Liu, Yanbin Zeng

**Affiliations:** State Key Laboratory of Electromechanical Integrated Manufacturing of High-Performance Electronic Equipments, Xidian University, Xi’an 710071, China

**Keywords:** variable stiffness gripper, modularity, parameter optimization, flexible drive

## Abstract

Soft grippers have good adaptability and flexibility for grasping irregular or fragile objects, and to further enhance their stiffness, soft grippers with variable stiffness have been developed. However, existing soft grippers with variable stiffness have the disadvantages of complex structure and poor interchangeability. Here, a soft gripper with modular variable stiffness is proposed that has flexible Velcro embedded in the bottom layer of the soft actuator and one side of the variable stiffness cavity respectively, and both the general and variable stiffness grasping modes are achieved by separation or combination. First of all, according to the neo-Hookean model and the assumption of constant curvature, a free bending model of the soft actuator is established and optimal structural parameters of the soft actuator are obtained by the Genetic Algorithm. Then, influence of the driving pressure on the soft actuator stiffness is investigated, and a mathematical model of the variable stiffness is established. Finally, correctness of the statics model and the stiffness model were verified by experiments. Experimental results indicate that the proposed soft gripper with modular variable stiffness structure has excellent adaptability and stability to different objects, outstanding load bearing capacity, and stiffness adjustment capability.

## 1. Introduction

Due to their naturally soft characteristics, soft robots may deform or chatter when performing certain tasks, therefore affecting their motion accuracy and stability. To improve their control precision, stiffness of the soft robots needs to be increased. Currently, there are two main methods to stiffen the soft robots: material stiffening and structural stiffening.

Shintake et al. [1] proposed a new variable stiffness actuator composed of a dielectric elastomer actuator (DEA) and a low-melting-point alloy (LMPA) embedded silicone substrate, where the DEA can generate a bending actuation and the LMPA can provide controllable stiffness by Joule heating. Firouzeh et al. [2] presented a tendon-driven robotic origami which can provide self-adaptability and inherent softness through its redundant and underactuated degrees of freedom. Based on a polymer layer with adjustable stiffness, stiffness for each joint can be controlled independently. Liu et al. [3] proposed a shape-memory alloy (SMA)-based soft gripper with variable stiffness composed of three robotic fingers for grasping compliantly at low stiffness and holding robustly at high stiffness, where the paraffin as a variable stiffness material in the joint can be heated or cooled to change the stiffness of the robotic fingers. Xiang et al. [4] designed an electro-adhesive gripper with variable stiffness and a simple construction based on low-melting-point alloys, where the active form adaptation and active changing stiffness can be achieved by pneumatic driving and resistance wires, respectively. Nishida et al. [5] developed a robot gripper based on an electromagnet and a reforming magnetorheological (MR) fluid which is employed to adjust the gripper stiffness. Haibin et al. [6] developed a variable stiffness mechanism for a soft robotic gripper with embedded sets of SMA fibers, and then implemented real-time computations of the grasping force based on Cosserat theory. The response characteristics of its backbone could comply well with the constant-curvature model. Yufei et al. [7] presented the design, fabrication, and function of a soft actuator embedded with low-melting-point alloy by melting the metal via Joule heating. The phase of the metal transformed from the solid state to the liquid state, by which the stiffness of the actuator changed over nearly an order of magnitude. Wang et al. [8] employed 4D electrohydrodynamic (EHD) printing to fabricate the highly deformable actuators with soft magnetic composites. This printing process offers a facile and effective path to fabricate soft magnetic composites toward potential applications. Cao et al. [9] proposed a soft electrothermal actuator with a microfilament heater embedded between a polyimide (PI) film and a polydimethylsiloxane (PDMS) layer, and the micro-heater was fabricated by the EHD printing process. Tang et al. [10] developed a high-performance soft EHD pump, enabling high-speed actuation and large deformation of untethered soft fluidic robots that have the capability of rapid large-area self-healing.

Inspired by muscular structures in the octopus, Shiva et al. [11] proposed a hybrid and inherently antagonistic actuation scheme for a soft manipulator, which is pneumatically actuated, and has tendons incorporated in the structure which complement the pneumatic actuation placed inside the manipulator’s wall to allow variation of overall stiffness. Brown et al. [12] demonstrated a universal robotic gripper based on the jamming of granular material, that is, individual fingers are replaced by a single mass of granular material that, when pressed onto a target object, flows around it and conforms to its shape. Wei et al. [13] combined a fiber-reinforced soft actuator and a particle flexible package to design an adaptive variable stiffness gripper, and the soft finger can be stiffened rapidly under vacuum pressure to resist external loads or to maintain the flexural shape of the soft finger.

Although particle blocking has attracted research interests in variable stiffness control, active particle blocking requires an additional negative pressure device which limits the portability of soft grippers. To address these issues, Li et al. [14] proposed a variable stiffness method based on passive particle obstruction which does not require any vacuum device or other control means. By filling the soft cavity with compressed gas it will drive the particles pack to bend passively, resulting in particle blockage inside the pack. Increasing the input air pressure will lead to tighter particle clogging, thus increasing the stiffness of the soft fingers. In addition, Li et al. [15] designed a distributed structure of the passive particle jamming soft gripper, which can increase the bending angle of an integral passive particle jamming gripper from 50° to 76° while roughly maintaining the gripper’s rotational stiffness and maximum pull-out forces. Jiang et al. [16] proposed a novel particle jamming mechanism based on the differential pressure drive, which is characterized by a dual-deformable chamber structure in which one chamber is filled with particles. Thus, the differential drive particle jamming mechanism can achieve the independent control of the stiffness and the bending angle.

In addition to particle obstruction, obstruction can also occur in laminar structures, Fang et al. [17] implemented a soft gripper with multiple grasping modes through a laminar obstruction structure and a tendon-driven mechanism with four grasping modes, including wrapping, pinching, hooking, and sucking. Zeng et al. [18] proposed a soft gripper based on layer interference technology. Layer interference plates are wrapped around a finger structure and then sealed inside a vacuum bag. When a heavy object needs to be grasped, the air inside the vacuum bag is extracted and the interference layers squeeze each other under air pressure, at which point the gripper locks into the actuated shape. Elgeneidy et al. [19] proposed a 3D printed soft gripper with layer interference capabilities. It was shown that layer jamming between the inclined flexible ribs of this soft hand gripper can enhance gripping forces at large displacements while maintaining minimal contact forces for fine grasping at small displacements. Chen et al. [20] designed a soft gripper with layer interference variable stiffness and electrostatic adsorption for enhancing the grasping performance. Wang et al. [21] proposed an electrostatic layer jamming variable stiffness technique for soft robotics, the basic principle of which is using electrostatic attraction to squeeze material layers to generate friction and engage jamming. Chen et al. [22] also proposed a negative pressure adsorption soft gripper based on layer interference variable stiffness.

In addition to the two variable stiffness modes mentioned above, the soft actuator is one of the most important components of soft robots, which can complete deformations such as bending, stretching, and twisting. Different actuators have different working mechanisms. Currently, the commonly used driving methods include hydraulic or pneumatic drive [23], cable drive [24], shape memory alloy (SMA) [25,26], shape memory polymer (SMP) [27], dielectric elastomer (DE) [28], ionic polymer–metal composites (IPMC) [29,30] and so on.

To summarize, many of the current variable stiffness soft grippers employ a monolithic design approach with a unique correspondence between the soft actuator and the soft gripper, which cannot be reconfigurable. Consequently, modular design of the soft gripper is a highly potential solution. Combining the properties of flexible materials and bionic principles, this manuscript proposed a soft gripper with modular variable stiffness structure, meaning that the deformable cavity and variable stiffness cavity are designed and fabricated separately, and then combined or separated by Velcro to achieve variable stiffness grasping or general grasping respectively.

The rest of this manuscript is organized as follows. In Section 2, structure of the soft actuator is designed. Finite element analysis and statics modeling of the soft actuator are investigated in Section 3 and Section 4. Stiffness of soft actuator is analyzed in Section 5, and in Section 6, the soft gripper is fabricated. Experiments including bending performance, stiffness test, and grasping performance are carried out in Section 7. Finally, Section 8 concludes the paper.

## 2. Actuator Design

**Figure 1:**
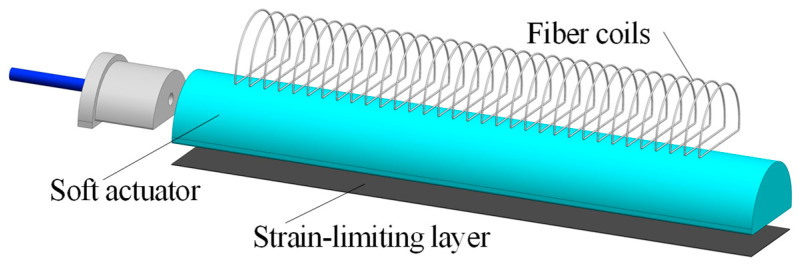
Structure of the soft actuator.

As shown in Figure 1, the first part of the soft actuator is a semi-cylindrical silicone chamber that can be filled with compressed air. The second part is a constraint layer, which is attached to the flat layer of the actuator and can convert the actuator elongation into bending deformation. The third part is a non-extendable coil that is employed to restrain the radial expansion of the silicone chamber so that the soft actuator can only elongate in the axis [31].

**Figure 2:**
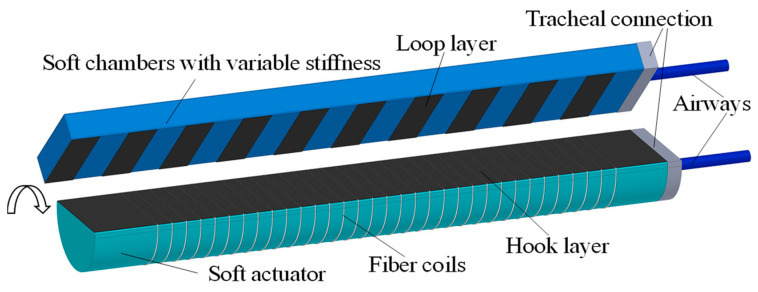
Soft actuator structure with variable stiffness.

The hook layer of the Velcro is used as the constraint layer of the soft actuator and the loop layer is used as the variable stiffness structure. The flexible connection between the modules is achieved by pasting or separating the hook layer and the loop layer, thus allowing for general or variable stiffness grasping. This innovative design can ensure the reliable and flexible connection between the actuator and the variable stiffness part. Structure of the modular soft actuator with variable stiffness is shown in Figure 2. To ensure that the variable stiffness structure does not wrinkle during bending, the loop layer is cut into strips and arranged evenly on one side of the variable stiffness structure.

**Figure 3:**
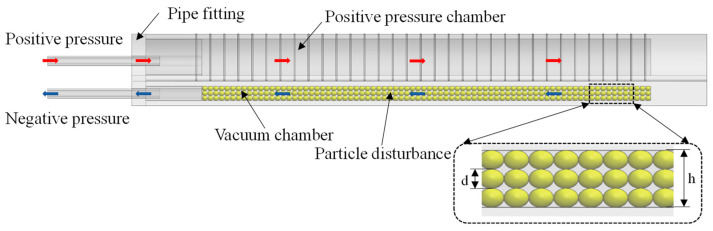
Working principle of the soft actuator with variable stiffness.

The working principle of the soft actuator with variable stiffness is shown in Figure 3. First of all, apply positive pressure to the corresponding chamber to bend it. At this moment, the vacuum chamber bend passively. When the actuator is bent to a certain extent, apply negative pressure to the negative pressure chamber, and stiffness of the actuator is increased under the effect of particle blockage.

**Figure 4:**
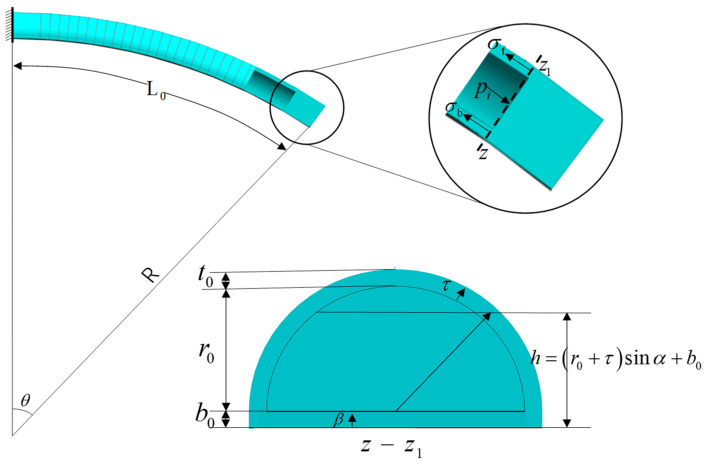
Soft actuators and cross-sections.

As shown in Figure 4, initial parameters of the soft actuator are set as: actuator length *L*_0_ = 200 mm, chamber radius *r*_0_ = 12 mm, flat layer thickness *b*_0_ = 2 mm, thickness of the semi-circular wall *t*_0_ = 3 mm, number of the coils *n* = (10, 15, 25, 35, 45).

## 3. Finite Element Analysis of Soft Actuator

**Figure 5:**
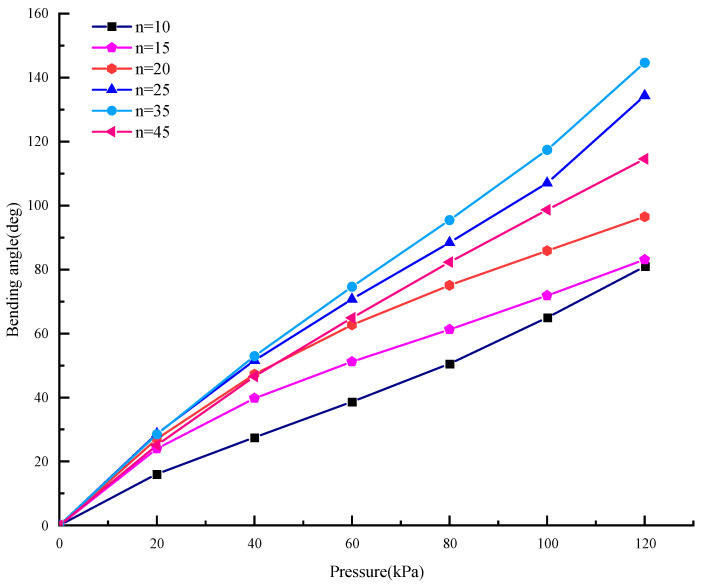
Bending angle of the actuators with different number of coils.

The effect of the coil turns on bending deformation of the soft actuator is shown in Figure 5. When the number of coils is too small (n ≤ 10), much of the area of the actuator will not be restrained and there will be large nonlinear protrusions and swelling instability under the pressure state. Obviously, the more the coil turns, the smaller the radial deformation of the soft actuator. When n ≥ 35, the radial deformation of the actuator can be ignored. Therefore, the optimal number of coils is chosen as 35.

**Figure 6:**
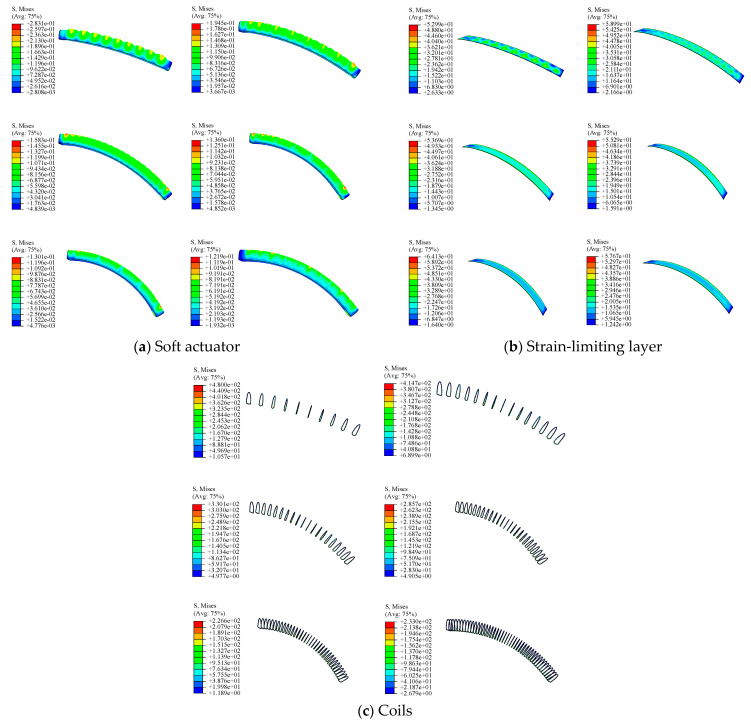
Stress clouds of each part at 60 kPa for different number of turns.

To analyze the stress distribution of the soft actuator, stress clouds of which under the constraint of different coil turns at 60 kPa was investigated. As shown in Figure 6a when the coil turns are small, average stress of the actuator is relatively high and the bending angle is smaller. That is because small number of coils cannot restrain the radial expansion of the actuator. As a result, axial deformation of the actuator will be smaller, that is, the bending angle is smaller. For n = 35, the maximum stress is mainly distributed on the semicircular wall of the actuator and distributed at both ends, which lays foundation for the actuator optimization in the following. Stress clouds of the strain limiting layer with different coil turns at 60 kPa are shown in Figure 6b at the same pressure. When n ≤ 20, the stress concentration is mainly symmetrically distributed along the centerline of the strain-limiting layer. When n ≥ 25, the stress distribution is more uniform, and the average stress is smaller. Stress clouds of the coil with different coil turns at 60 kPa is shown in Figure 6c—the more the coil turns, the smaller the average stress.

## 4. Statics Modeling of Soft Actuator

Statics model of the soft actuator in a free bending state is shown in Figure 4. It is assumed that the actuator conforms to the constant curvature model during the bending process and the strain-limiting layer is not stretchable. Then, the balance of moments around the *z*-*z*_1_ axis gives:

(1) $$M_{P}=M_{b}+M_{t}$$

where *M*_P_ is the moment generated by the driving pressure, *M*_t_ is the elastic moment generated by the semicircular wall of the soft actuator, and *M*_b_ is the elastic moment generated by the rectangle at the bottom of the soft actuator. *M*_P_ can be expressed as:

(2) $$M_{P}=\int_{0}^{\frac{\pi }{2}}\left(r_{0}\sin\alpha +b_{0}\right)dF$$

During the bending process, the stretch ratio is assumed to be 1 at the neutral axis, that is, at the fabric layer, and the stretch ratio varies linearly along the soft actuator thickness.

(3) $$\lambda_{b}=1+\beta \theta /L_{0}$$

(4) $$\lambda_{t}=1+(b_{0}+(r_{0}+\tau )\sin\alpha )\theta /L_{0}$$

where, *λ*_t_ and *λ*_b_ are the stretching ratios of the soft actuator at the semicircular wall and bottom, respectively. Here, nominal axial stress in the incompressible material can be solved using *s_i_* = (*λ_i_* − *λ_i_*^−3^), where *μ* is the corresponding shear modulus in the N-H model. Combining (3) and (4), *M*_b_ and *M*_t_ can be obtained as:

(5) $$M_{b}=2\mu (r_{0}+t_{0})\int_{0}^{b_{0}}\left((1+\beta \theta /L_{0})-{\left(1+\beta \theta /L_{0}\right)}^{-3}\right)\beta d\beta$$

(6) $$\begin{array}{ll} M_{t} & =\mu \int_{0}^{t_{0}}\int_{0}^{\pi }\left(1+(b_{0}+(r_{0}+\tau )\sin\alpha )\theta /L_{0}\right)[(r_{0}+\tau )2\sin\alpha +b_{0}(r_{0}+\tau )]d\alpha d\tau \\ & -\mu \int_{0}^{t_{0}}\int_{0}^{\pi }{\left(1+(b_{0}+(r_{0}+\tau )\sin\alpha )\theta /L_{0}\right)}^{-3}[(r_{0}+\tau )2\sin\alpha +b_{0}(r_{0}+\tau )]d\alpha d\tau \end{array}$$

To simplify the equation, the following series expansion can be employed:

(7) $${\left(1+q\right)}^{-3}=1-3q+\frac{3\times 4}{2!}q^{2}-\ldots$$

Thus, the simplified elastic moments *M*_b_ and *M*_t_ can be expressed as:

(8) $$M_{b}=2\mu (r_{0}+t_{0})(4b_{0}^{3}\theta /3L_{0}-3b_{0}^{4}\theta^{2}/2L_{0}^{2})$$

(9) $$\begin{array}{ll} M_{t} & =\mu \int_{0}^{t_{0}}\int_{0}^{\pi }\left[4(b_{0}+(r_{0}+\tau )\sin\alpha )\theta /L_{0}\right][(r_{0}+\tau )2\sin\alpha +b_{0}(r_{0}+\tau )]d\alpha d\tau \\ & -\mu \int_{0}^{t_{0}}\int_{0}^{\pi }\left[6{\left(b_{0}+(r_{0}+\tau )\sin\alpha \right)}^{2}\theta^{2}/L_{0}^{2}\right][(r_{0}+\tau )2\sin\alpha +b_{0}(r_{0}+\tau )]d\alpha d\tau \end{array}$$

Thus, the relationship between air pressure and bending angle can be expressed as:

(10) $$P(\theta )=k_{1}\theta +k_{2}\theta^{2}$$

where, *k*_1_ and *k*_2_ are:

(11) $$k_{1}=\mu \frac{4(r_{0}+t_{0})b_{0}^{3}+6\int_{0}^{t_{0}}\int_{0}^{\pi }(b_{0}+(r_{0}+\tau )\sin\alpha )({\left(r_{0}+\tau \right)}^{2}\sin\alpha +b_{0}(r_{0}+\tau ))d\alpha d\tau }{3r_{0}^{2}L_{0}(\pi b_{0}/4+r_{0}/3)}$$

(12) $$k_{2}=\mu \frac{-3(r_{0}+t_{0})b_{0}^{4}-6\int_{0}^{t_{0}}\int_{0}^{\pi }{\left(b_{0}+(r_{0}+\tau )\sin\alpha \right)}^{2}({\left(r_{0}+\tau \right)}^{2}\sin\alpha +b_{0}(r_{0}+\tau ))d\alpha d\tau }{2r_{0}^{2}L_{0}^{2}(\pi b_{0}/4+r_{0}/3)}$$

Then, the angle *θ* can be solved inverse as a function of *P* as follows:

(13) $$\theta =f^{-1}(P)=\left(-k_{1}+\sqrt{k_{1}^{2}+4k_{2}P}\right)/2k_{2}$$

The maximum bending angle is taken as the optimization objective for the structural parameters of the soft actuator and, based on (13), the objective function is chosen as:

(14) $$\min\;\frac{1}{\theta }=\frac{2k_{2}}{-k_{1}+\sqrt{k_{1}^{2}+4k_{2}}}$$

(15) $$s.t.\;\left\{\begin{array}{l} 2.5\leq t_{0}\leq 4\\ 10\leq r_{0}\leq 15\\ 3\leq b_{0}\leq 4.5\\ 1<b_{0}/t_{0}\leq 2.5\\ 15\leq r_{0}+t_{0}\leq 20 \end{array}\right.$$

where, *k*_1_ and *k*_2_ can be derived from (11) and (12), respectively. The minimum thickness of the actuator is set to 2 mm. To ensure the strength of the soft actuator, the minimum wall thickness is set to 2.5 mm and the maximum wall thickness is set to 4 mm. The minimum and maximum thickness of the actuator is set to 3 mm and 4.5 mm, respectively. To ensure the sufficient strength, *b*_0_/*t*_0_ is greater than 1 and less than or equal to 2.5 [32]. The bottom width of the actuator is set from 30 mm to 40 mm, so *r*_0_ + *t*_0_ is between 15 mm and 20 mm. Structural parameters of the actuator optimized by the Genetic Algorithm are shown in Table 1.

**Figure 7:**
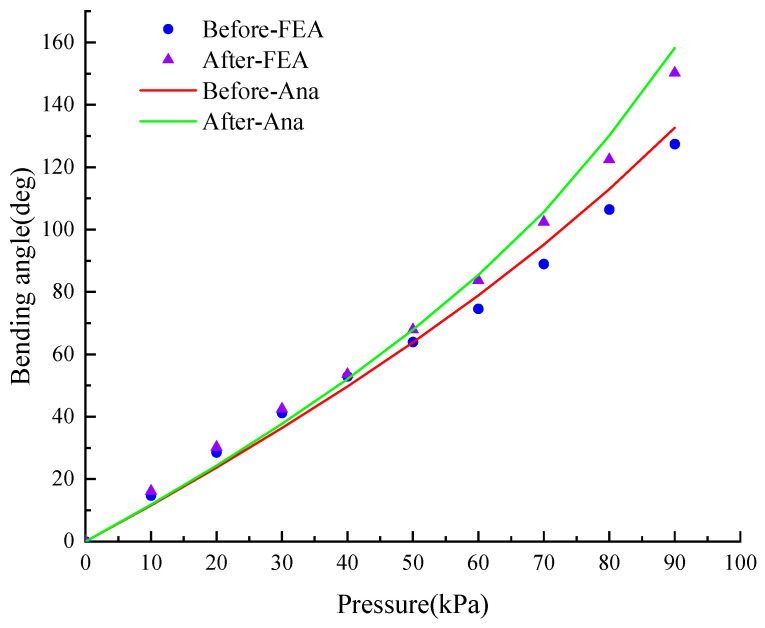
Comparison of bending angles before and after structural optimization.

From Figure 7, at the same pressure, when *P* ≤ 50 kPa, the free bending angle of the optimized actuator is only slightly larger than that of the unoptimized one. When *P* > 50 kPa, the optimized bending angle is significantly larger than that of the unoptimized one. For nine sets of data under different air pressures, the root means square error between the analytical solution and the finite element analysis (FEA) is 4.8805°.

## 5. Stiffness Analysis of Soft Actuator

**Figure 8:**
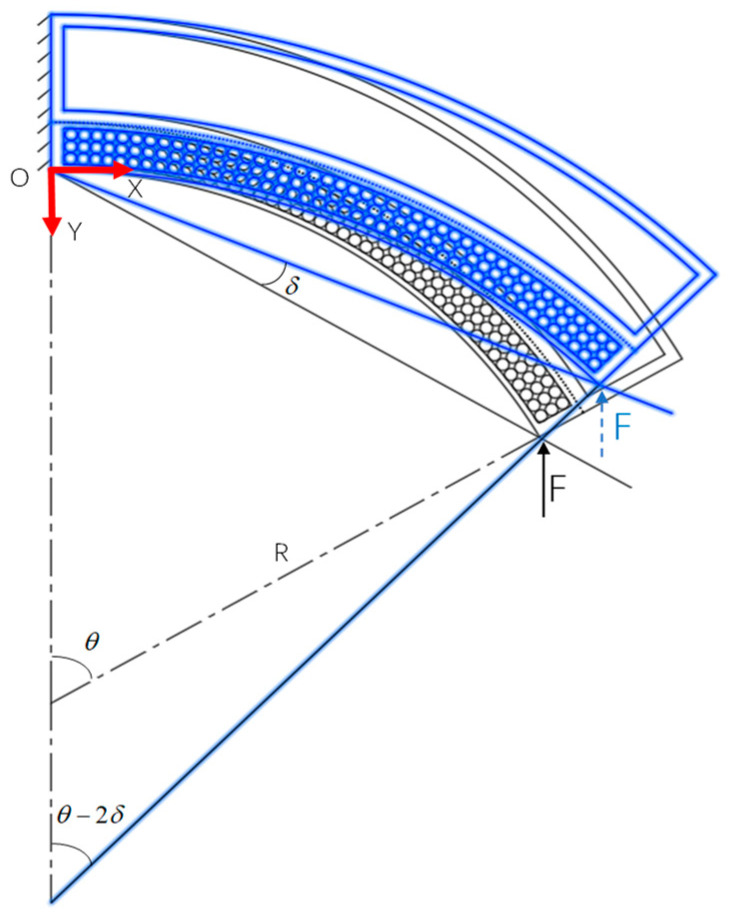
Stiffness analysis of the variable stiffness soft actuator.

**Figure 9:**
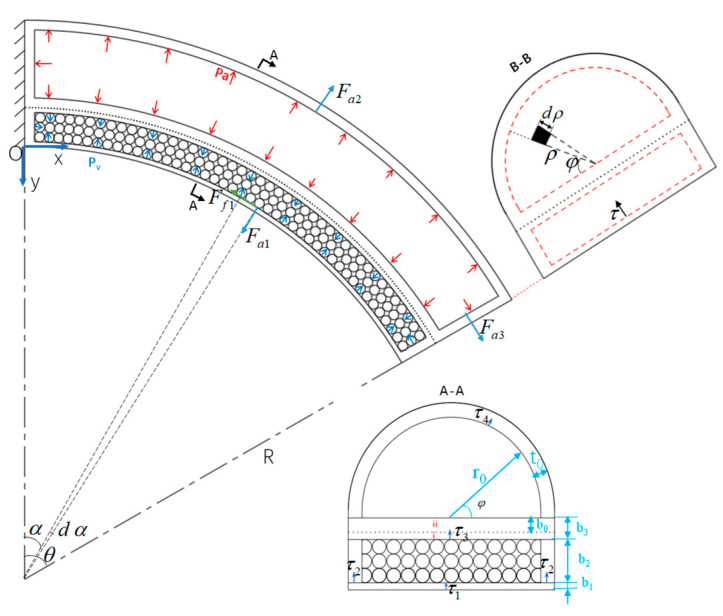
Force analysis of the variable stiffness soft actuator.

Stiffness of the soft actuator with variable stiffness is defined as the rotational stiffness as shown in Figure 8. When the actuator is bent to *θ* under air pressure, an external force *F* is then applied to its end to simulate the load which causes the actuator to rotate an angle *δ* around the point *O*. The rotational stiffness is then defined as:

(16) $$K=M_{F}\delta^{-1}$$

where *M*_F_ is the applied torque and *δ* is the rotation angle of the soft actuator. The external torque *M*_F_ = *FR*sin*θ* can be calculated by experiments. Stiffness of the soft actuator is modelled theoretically by balancing moments such as load torque and drive torque, and the following assumptions are made:

(1) When the fiber reinforced actuator bends the variable stiffness structure passively, assuming that the loop layer of the variable stiffness structure maintains close contact with the hook layer of the fiber reinforced actuator without slip.
(2) Based on assumption (1), bottom (ii) of the soft actuator and top (i) of the variable stiffness structure are assumed to be one and the same when calculating the moments of the variable stiffness soft actuator, as shown in the A–A partial diagram in Figure 9.
(3) It is assumed that the particles are incompressible, and their weight is negligible.

Where *P*_a_ represents the positive pressure acting on the actuator and *P*_v_ is the negative pressure acting on the particle cavity, and both are the air pressure values relative to atmospheric pressure. As shown in Figure 8, the actuator is mainly subject to the particle interference moment *M*_k_, the air pressure driving moment *M*_a_, the elastic moment *M*_e_, and the external load moment *M*_F_, where *M*_a_ bends the actuator and *M*_k_ inhibits the actuator from bending in the opposite direction when subjected to external forces. Both *M*_e_ and *M*_F_ can prevent the actuator from bending. Therefore, the equation for the moment balance of the soft actuator at point *O* can be expressed as:

(17) $$M_{a}+M_{k}=M_{e}+M_{F}$$

### 5.1. Disturbance Moment of the Particles

Disturbance moment *M*_k_ of the particles is caused by the friction between the particles and the internal surface of the variable stiffness cavity. Here, the force transfer between particles is simplified to a fluid transfer process, where the vacuum pressure is equivalently transferred to the inner wall of the variable stiffness cavity by squeezing the particles. Seen from Figure 9, the friction *F*_f1_ generated between the particles and the inner wall of the particle cavity is oriented perpendicular to the bending radius of the variable stiffness structure. A micro-element d*S* can be selected on its inner surface and d*S* = 2*r*_0_(*L*_0_/*θ* + *b*_1_)d*α* so that the frictional moment at the lower surface inside the particle cavity can be obtained as:

(18) $$M_{k1}=2\mu_{f}P_{s}r_{0}\int_{0}^{\theta }\left[{\left(L_{0}/\theta +b_{1}\right)}^{2}\sin^{2}\alpha -(L_{0}/\theta +b_{1})(L_{0}/\theta -(L_{0}/\theta +b_{1})\cos\alpha )\cos\alpha \right]d\alpha$$

where, *P*_s_ = *P*_a_ + |*P*_v_| and *μ*_f_ is the friction coefficient between the particles and the cavity surface. Similarly, the frictional moment on both sides of the cavity surface can be obtained as:

(19) $$M_{k2}=2\mu_{f}P_{s}b_{2}\int_{0}^{\theta }\left({\left(L_{0}/\theta +b_{1}+b_{2}/2\right)}^{2}\sin^{2}\alpha -(L_{0}/\theta -(L_{0}/\theta +b_{1}+b_{2}/2)\cos\alpha )\cos\alpha \right)d\alpha$$

The frictional moment on the upper surface of the particle cavity is:

(20) $$\begin{array}{ll} M_{k3} & =\mu_{f}P_{s}\int_{0}^{\theta }2r_{0}{\left(L_{0}/\theta +b_{1}+b_{2}\right)}^{2}\sin^{2}\alpha d\alpha \\ & -\mu_{f}P_{s}\int_{0}^{\theta }2r_{0}(L_{0}/\theta +b_{1}+b_{2})(L_{0}/\theta -(L_{0}/\theta +b_{1}+b_{2})\cos\alpha )\cos\alpha d\alpha \end{array}$$

Therefore, the total moment generated by the particle disturbance is:

(21) $$M_{k}(\theta ,P_{a},P_{v})=M_{k1}+M_{k2}+M_{k3}$$

### 5.2. Moment of the Air Pressure

The moment generated by air pressure after the actuator bends is mainly composed of the inner surface of the variable stiffness structure, the top semicircular layer of the fiber reinforced actuator, and the moment on the end cover. Considering the air pressure in *x*-direction is very small and can be neglected. Therefore, the moment acting on the inner side is:

(22) $$M_{a1}=\int_{0}^{\theta }2r_{0}P_{s}{\left(L_{0}/\theta +b_{1}\right)}^{2}\cos\alpha \sin\alpha d\alpha$$

Pressure acting on the semicircular wall produces a moment at point *O*.

(23) $$M_{a2}=-\int_{0}^{\theta }\int_{0}^{\pi }P_{a}r_{0}{\left(L_{0}/\theta +b_{1}+b_{2}+b_{3}+r_{0}\sin\varphi \right)}^{2}\cos\alpha \sin\alpha \sin\varphi d\alpha d\varphi$$

In order to study the moment generated by the force *F*_a3_ acting on the end face of the actuator, the local coordinates *ρ*, *φ,* and *τ* are introduced at the end face B–B of the soft actuator to obtain the moment of the air pressure on the end face of the soft actuator for the point O.

(24) $$\begin{array}{ll} M_{a3} & =\int_{0}^{r_{0}}\int_{0}^{\pi }P_{a}\rho (\rho \sin\varphi +L_{0}/\theta +b_{1}+b_{2}+b_{3})\sin^{2}\theta d\varphi d\rho \\ & -\int_{0}^{r_{0}}\int_{0}^{\pi }P_{a}\rho (L_{0}/\theta -(L_{0}/\theta +b_{1}+b_{2}+b_{3}+\rho )\cos\theta )\cos\theta d\varphi d\rho \\ & +\;2r_{0}P_{v}\int_{0}^{b_{2}}\left((L_{0}/\theta +b_{1}+\tau )\sin^{2}\theta -(L_{0}/\theta -(L_{0}/\theta +b_{1}+\tau )\cos\theta )\cos\theta \right)d\tau \end{array}$$

Therefore, the total moment produced by air pressure on the variable stiffness soft actuator is:

(25) $$M_{a}(\theta ,P_{a},P_{v})=M_{a1}+M_{a2}+M_{a3}$$

### 5.3. Moment of the Elastic Stress

As shown in the A–A section of Figure 9, to facilitate the investigation of deformation stresses in various parts of the actuator, it is divided into four parts. The local coordinate systems *τ*_1_, *τ*_2_, *τ*_3_, and *τ*_4_ are established in these four parts, and the elongation along the axial direction of these four parts are:

(26) $$\left\{\begin{array}{l} \lambda_{\tau_{1}}=\left(L_{0}+\tau_{1}\theta \right)/L_{0}\\ \lambda_{\tau_{2}}=\left[L_{0}+(b_{1}+\tau_{2})\theta \right]/L_{0}\\ \lambda_{\tau_{3}}=\left[L_{0}+(b_{1}+b_{2}+\tau_{3})\theta \right]/L_{0}\\ \lambda_{\tau_{4}}=[L_{0}+(b_{1}+b_{2}+b_{3}+(r_{0}+\tau_{4})\sin\varphi )]\theta /L_{0} \end{array}\right.$$

where *λ_τ_*_1_, *λ_τ_*_2_, *λ_τ_*_3_, and *λ_τ_*_4_ are the axial tension ratios of the bottom, the side, the middle, and the top semicircular layers of the actuator. Their stresses can be calculated using *σ_i_* = *μ*(*λ_i_* − *λ_i_*^−3^). The torques generated by the elastic deformation of these four components to the point *O* are:

(27) $$\left\{\begin{array}{l} M_{e1}=\int_{0}^{b_{1}}2\sigma_{1}(r_{0}+t_{0})\tau_{1}d\tau_{1}\\ M_{e2}=2\int_{0}^{b_{2}}\sigma_{2}b_{2}(b_{1}+\tau_{2})d\tau_{2}\\ M_{e3}=\int_{0}^{b_{3}}2\sigma_{3}(r_{0}+t_{0})(b_{1}+b_{2}+\tau_{3})d\tau_{3}\\ M_{e4}=\int_{0}^{t_{0}}\int_{0}^{\pi }\sigma_{4}((r_{0}+\tau_{4})\sin\varphi +b_{1}+b_{2}+b_{3})(r_{0}+\tau_{4})d\varphi d\tau_{4} \end{array}\right.$$

Therefore, the torque of the variable stiffness soft actuator at the point *O* is:

*M*_e_ = *M*_e1_ + *M*_e2_ + *M*_e3_ + *M*_e4_ (28)

According to the stiffness definition in (16), stiffness *K*_b_ of the soft actuator with variable stiffness structure is:

(29) $$K_{b}=[M_{a}(\theta -2\delta ,P_{a},P_{v})+M_{k}(\theta -2\delta ,P_{a},P_{v})-M_{e}(\theta -2\delta )]\delta^{-1}$$

When calculating the theoretical stiffness of the soft actuator, (29) can be corrected. Due to the soft actuator has no particle disturbance moment, *M*_k_ = 0. The soft actuator only has positive pressure cavities and is not subject to negative pressure, meaning *P*_v_ = 0. Considering the absence of a variable stiffness cavity in the fiber reinforced actuator, *M*_e1_ = *M*_e2_ = 0. Therefore, the elastic torque generated by the soft actuator at point *O* is:

(30) $$\begin{array}{ll} M_{e}^{\prime } & =\int_{0}^{b_{0}}2\sigma_{3}(r_{0}+t_{0})(b_{1}+b_{2}+b_{3}-b_{0}+\tau_{3})d\tau_{3}\\ & +\int_{0}^{t_{0}}\int_{0}^{\pi }\sigma_{4}((r_{0}+\tau_{4})\sin\varphi +b_{1}+b_{2}+b_{3})(r_{0}+\tau_{4})d\varphi d\tau_{4} \end{array}$$

Thus, the theoretical stiffness *K*_x_ of the general soft actuator is:

(31) $$K_{x}=[M_{a}(\theta -2\delta ,P_{a},0)-M_{e}^{\prime }(\theta -2\delta )]\delta^{-1}$$

## 6. Gripper Fabrication

**Figure 10:**
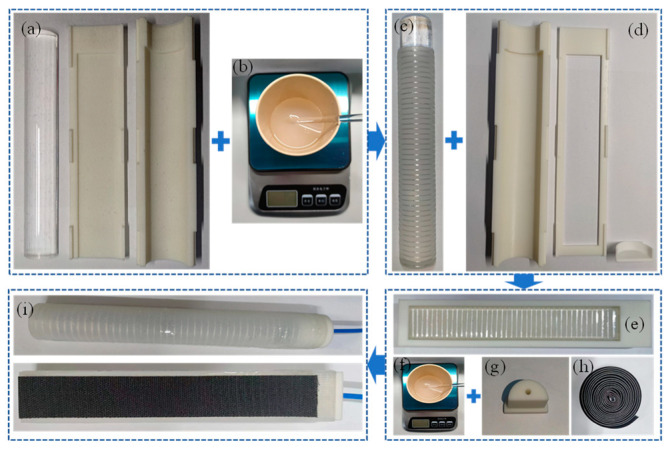
Fabrication process of the soft actuator. (**a**) Core and mold. (**b**) Silicone solution. (**c**) Basic structure. (**d**) Mold. (**e**) Assembly structure. (**f**) Silicone solution. (**g**) Pipe interface. (**h**) Hook layer. (**i**) Soft actuator.

Here, the soft actuator is made using the pour and form method. Physical fabrication of the soft actuator is shown in Figure 10. Firstly, the base of the soft actuator is poured and then the coils are wound on its surface and a strain limiting layer is added to its bottom. Finally, the base surface is poured once more. Figure 10a,d are the first and second casting of the mold respectively, and Figure 10i shows the completed soft actuator.

**Figure 11:**
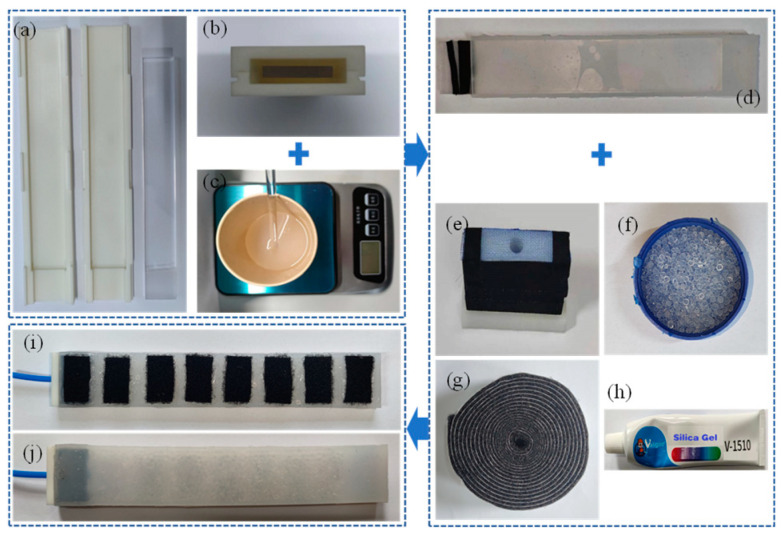
Fabrication process of the variable stiffness structure. (**a**) Mold. (**b**) Assembly of mold and core. (**c**) Silicone solution. (**d**) Soft cavity. (**e**) Pipe interface. (**f**) Particles. (**g**) Loop layer. (**h**) Silicone adhesive. (**i**) Variable stiffness structure. (**j**) The other side.

Physical fabrication of the variable stiffness structure is shown in Figure 11. The loop layer shown in Figure 11i can be adhered to the hook side in Figure 10h to form a complete variable stiffness soft actuator. Figure 11j shows the other side of the variable stiffness structure, which is mainly in contact with the surface of the object to be grasped and has the advantage of adaptive grasping due to its softness and variable stiffness.

**Figure 12:**
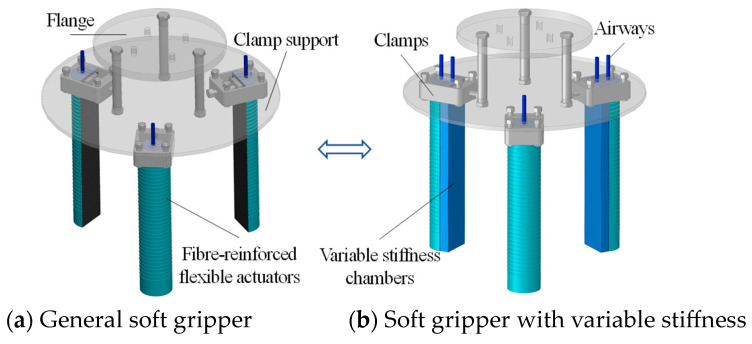
Assembly of modular variable stiffness soft grippers.

Assembly of the modular variable stiffness soft gripper is shown in Figure 12, where the general soft gripper in Figure 12a can be used to grip lighter masses and the soft gripper with variable stiffness in Figure 12b can be used to grip heavier masses.

## 7. Experiments

### 7.1. Bending Performance

**Figure 13:**
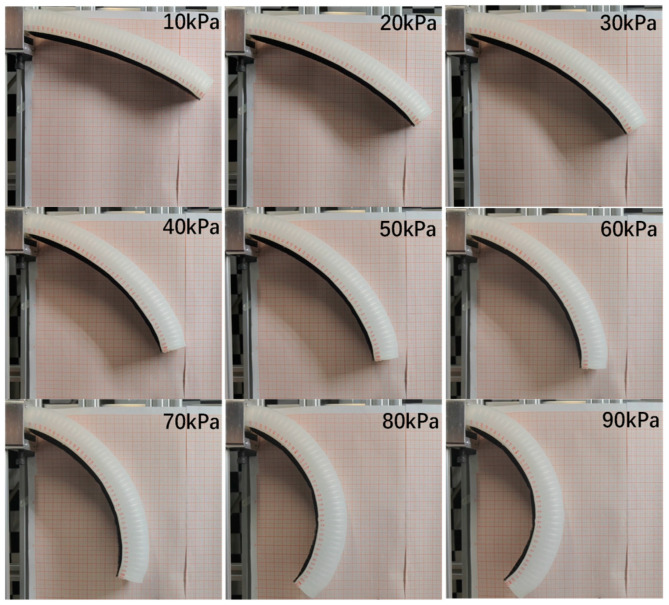
Bending angle at different air pressures.

From Figure 13, the bending angle of the soft actuator increases with the increasing of the air pressure. However, the relationship between the bending angle of the soft actuator and the air pressure is nonlinear, and between 70–90 kPa the bending angle of the soft actuator increases exponentially.

**Figure 14:**
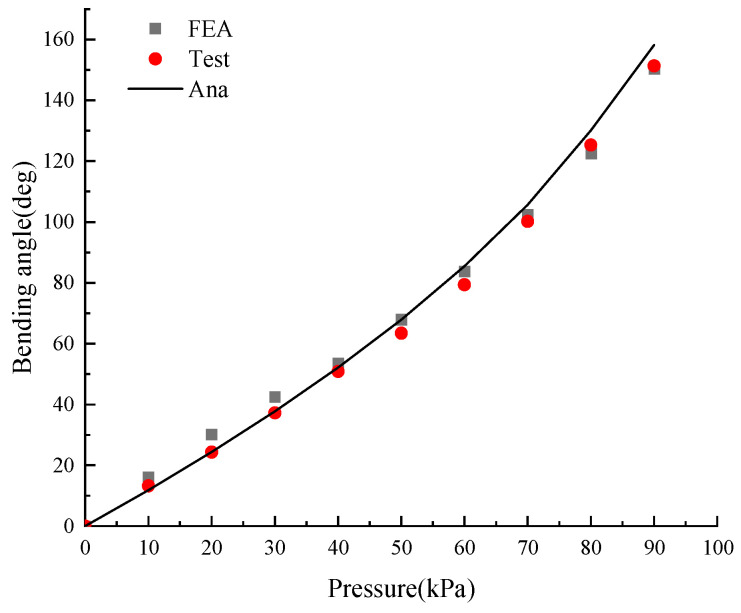
Relationship between bending angle and air pressure. (general soft gripper).

In Figure 14, the experimentally tested bending angle is compared with that of the finite element analysis (FEA) and theoretical calculation (Ana) respectively, and *θ*_Test_ has a root mean square error (RMSE) of 3.8246° and 4.2037° with respect to *θ*_FEA_ and *θ*_Ana_. Experiments show that the difference between the test value and the theoretical value is quite small, which verifies the correctness of the statics model of the soft actuator.

**Figure 15:**
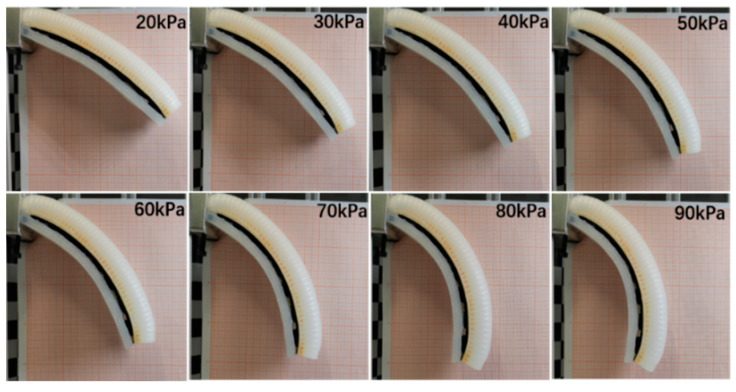
Bending angles at different air pressures.

**Figure 16:**
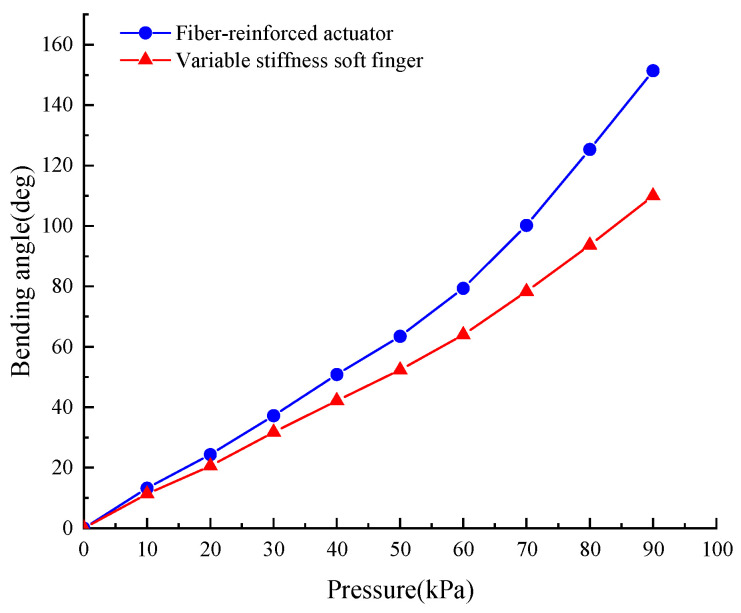
Relationship between bending angle and air pressure. (with variable stiffness).

As shown in Figure 15 and Figure 16, the bending angle of the soft actuator with the variable stiffness structure is smaller than that of the soft actuator under the same air pressure, and the difference in bending angle between these two actuators increases with the increasing of air pressure. It is obvious that the variable stiffness structure can prevent the soft actuator from bending. As the air pressure increases, obstruction of the particles in the stiffening structure becomes more pronounced. However, the soft actuator with variable stiffness bends well under 70–90 kPa, which provides a basis for grasping various objects.

### 7.2. Stiffness Test

**Figure 17:**
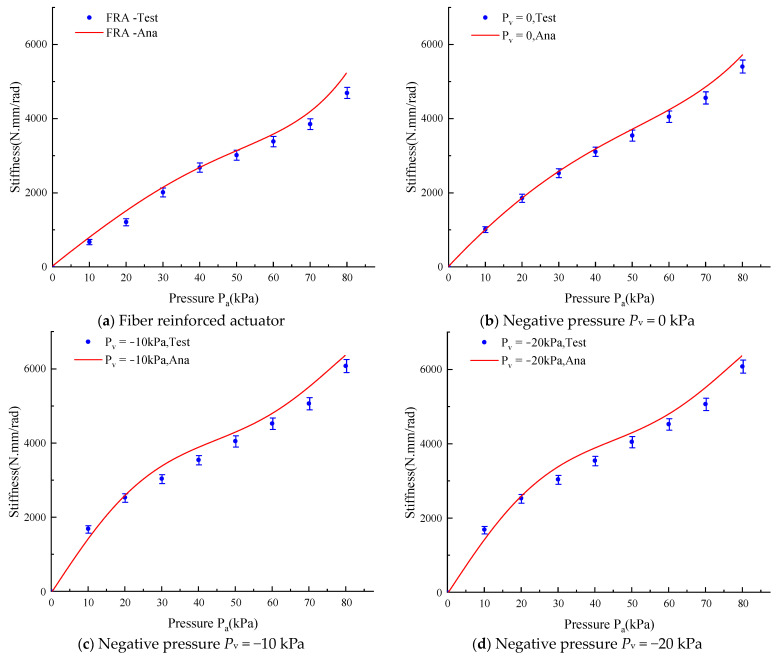
Experimental versus theoretical stiffness of the soft actuator.

As for the soft actuator stiffness, comparison of the experiments with the theoretical calculation is shown in Figure 17. It can be seen that the proposed stiffness model for the soft actuator with variable stiffness is effective in predicting the stiffness change with air pressure changes. However, the experimental results are slightly smaller than that of the theoretical calculations, possibly because the elastic force of strain limiting layer in the soft actuator is neglected.

As for the effect of particle size or type on the stiffness of the soft actuator, we have not conducted relevant experiments. However, qualitative analysis has shown that, on the one hand, the smaller the particle volume, the higher the filling density of the cavity, which results in a higher actuator stiffness. On the other hand, the smoother the surface and more regular the shape of the particles, the smaller the friction coefficient of the particles in contact with each other, which results in a smaller actuator stiffness.

**Figure 18:**
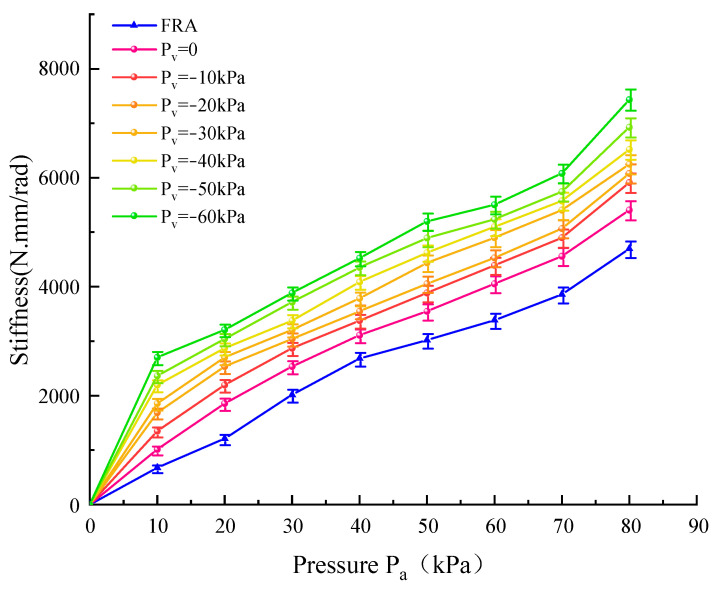
Relationship between stiffness and air pressure.

From Figure 18, under the same *P*_a_, stiffness of the soft actuator with variable stiffness is greater than that of the fiber reinforced actuators, even without negative pressure. This is because the variable stiffness structure bends passively and causes the particles blockage, thereby improving the stiffness of the entire soft actuator. In addition, the stiffness of both the soft actuator and the variable stiffness soft actuator increases steadily as *P*_a_ increases. Under the same *P*_a_, stiffness of the actuator with variable stiffness increases with the negative pressure increasing, and the area between the curve *P*_v_ = 0 and the *P*_v_ = −60 kPa is roughly the range of adjustable stiffness. This indicates that the adjustment range of stiffness of the modular variable stiffness soft actuator is relatively large, and allows the flexible use of the soft grippers for different grasping tasks.

### 7.3. Grasping Performance

**Figure 19:**
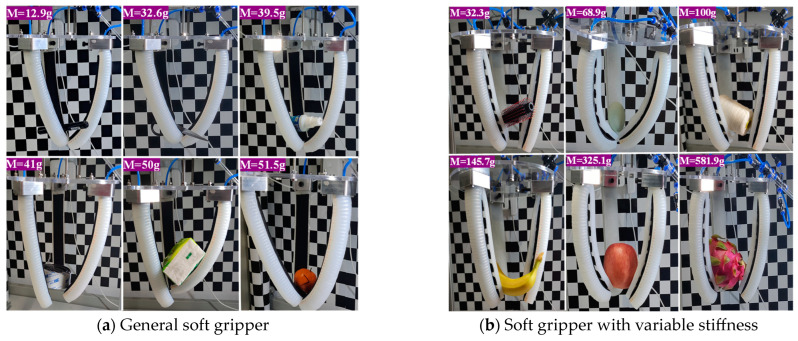
Grasping performance experiments.

Experiments for grasping different objects with the general soft gripper is shown in Figure 19a, which has a fast and flexible response. However, limited stiffness prevents it from grasping heavier objects. In Figure 19b, grasping experiments of the soft gripper with variable stiffness is carried out in terms of the load-bearing capacity, the grasping weight of which is much greater than that of the general soft gripper. However, the structure of the soft gripper with variable stiffness is quite cumbersome and it is difficult to accurately grasp for some smaller objects.

**Figure 20:**
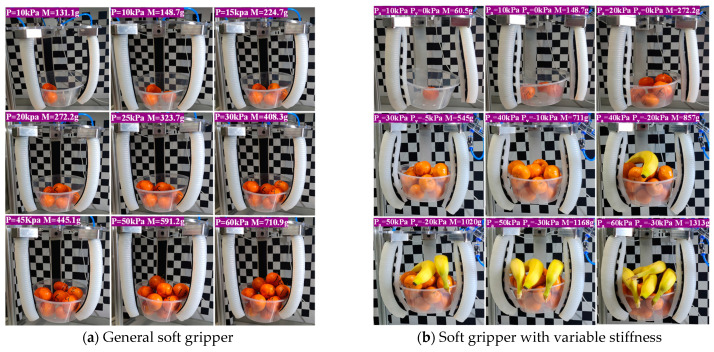
Load-bearing experiment.

To verify the load-bearing capacity, firstly, a given air pressure is applied to the soft gripper and the calibrated objects are gradually placed into the container until the container begins to detach from the gripper. At this moment, the weight of the entire container is the maximum load-bearing weight of the soft gripper under the given air pressure. Figure 20a is the load-bearing capacity testing process of the general soft gripper. As the air pressure increases, the maximum load-bearing capacity increases, and as the air pressure increases, so does its own stiffness. From Figure 20a, the maximum grasping weight of the general soft gripper is 710.9 g at 60 kPa air pressure.

As shown in Figure 20b, the load-bearing capacity of the soft gripper with variable stiffness is much higher than that of the general soft gripper. At a positive pressure of *P*_a_ = 60 kPa and *P*_v_ = −30 kPa, the maximum grasping weight of the soft gripper with variable stiffness can reach 1313 g, approximately 1.84 times the load-bearing capacity of a general soft gripper.

## 8. Conclusions

Considering the complex structure, poor interchangeability, and other disadvantages in existing soft grippers with variable stiffness structure, a novel soft gripper with the modular variable stiffness is proposed. This not only simplifies the manufacturing difficulty but also possesses both general and variable stiffness grasping modes simultaneously. Firstly, based on FEA, optimal number of the coil turns is obtained, meanwhile optimal structural parameters of the soft actuator are obtained by the Genetic Algorithm. Secondly, factors affecting the stiffness of soft actuator are investigated and a mathematical model for characterizing the stiffness of soft actuator is established. Thirdly, correctness of the bending model is verified through the free bending experiment of soft actuator, rationality of the designed soft actuator is verified through bending performance experiments, and the stiffness test shows that the proposed soft actuator has a large range of variable stiffness. Finally, by grasping various objects with different structures and materials, the adaptability and flexibility of the soft gripper has been verified, and the load-bearing capacity of the soft gripper with variable stiffness module is much better than that of the general soft gripper.

## Figures and Tables

**Table 1:** Optimized structural parameters for soft actuators.

Parameters	*L*_0_/mm	*t*_0_/mm	*b*_0_/mm	*r*_0_/mm	*n*
Values	200	2.5	3	15	35

## Data Availability

The data presented in this study are available on request from the corresponding author. The data are not publicly available due to the extremely large size.
